# To intubate or to resuscitate: the effect of simulation-based training on advanced airway management during simulated paediatric resuscitations

**DOI:** 10.1186/s41077-024-00326-y

**Published:** 2025-01-06

**Authors:** C. Donath, A. Leonhardt, T. Stibane, S. Weber, N. Mand

**Affiliations:** 1 Neonatology and Paediatric Intensive Care Medicine, Department of Paediatrics, Philipps-University Marburg, Marburg, Germany; 2 Dr. Reinfried-Pohl-Zentrum for Medical Learning, Philipps-University Marburg, Marburg, Germany; 3 General Paediatrics, Paediatric Nephrology and Transplant Nephrology, Department of Paediatrics, Philipps-University Marburg, Marburg, Germany

**Keywords:** Paediatric resuscitation, Simulation training, Airway management, Chest compressions

## Abstract

**Background:**

We aimed to measure the effect of a 2-day structured paediatric simulation-based training (SBT) on basic and advanced airway management during simulated paediatric resuscitations.

**Methods:**

Standardised paediatric high-fidelity SBT was conducted in 12 of the 15 children’s hospitals in Hesse, Germany. Before and after the SBT the study participants took part in two study scenarios (PRE and POST scenario), which were recorded using an audio–video system. Airway management was assessed using a performance evaluation checklist. Time to initiate ventilation, frequency, and timing of endotracheal intubation (ETI), and its influence on other life support interventions were assessed. Differences in airway management between hospitals with and without a PICU were evaluated.

**Results:**

Two hundred twenty-nine participants formed 58 interprofessional resuscitation teams. All teams recognised apnoea in their simulated patients and initiated ventilation during the scenarios. Time to recognition of apnoea and time to initiation of ventilation did not improve significantly after SBT, but teams were significantly more likely to select appropriately sized airway equipment. ETI was attempted in 55% PRE and 40% POST scenarios (*p*=0.1). The duration of the entire ETI process was significantly shorter in the POST scenarios. Chest compressions (CC) were frequently discontinued during ETI attempts, which improved after SBT (PRE 73% vs. POST 43%, *p* = 0.035). Adequate resumption of CC after completion of intubation was also significantly more frequent in the POST scenarios (46% vs. 74%, *p* = 0.048). During ETI attempts, CC were more likely to be adequately continued in teams from hospitals with a PICU (PRE scenarios: PICU 20% vs. NON-PICU 36%; POST scenarios: PICU 79%, NON-PICU 22%; *p* < 0.01).

**Conclusions:**

Our data suggest an association between airway management complexity and basic life support measures. Although the frequency of ETI was not significantly reduced after a 2-day SBT intervention, the duration of advanced airway management was shortened thus reducing no-ventilation time which led to fewer interruptions in chest compressions during simulated paediatric resuscitations. SBT may be adapted to the participants’ workplace to maximize its effect and improve the overall performance in paediatric resuscitation.

**Supplementary Information:**

The online version contains supplementary material available at 10.1186/s41077-024-00326-y.

## Introduction

Paediatric cardiac arrest (CA) is a rare event both in and out of the hospital. Even in larger hospitals with paediatric intensive care units (PICU), the average number of cardiopulmonary resuscitations (CPR) per month is less than one [1, 2]. Out-of-hospital cardiac arrest (OHCA) is even rarer. Data from the German Resuscitation Registry showed a yearly incidence of 3 OHCA per 100,000 children [3]. Due to the limited number of cases, healthcare professionals struggle to develop a sufficient routine when dealing with paediatric emergencies and resuscitations, possibly reducing the quality of care provided [4–6].

Despite ongoing efforts to improve the quality of resuscitation and post-resuscitation care, survival rates, particularly for OHCA, remain low and the neurological outcome of survivors is poor [3, 7–10] with only 5–24% of OHCA survivors showing no change in neurological status at discharge [11, 12]. In-hospital cardiac arrest (IHCA) commonly occurs in chronically ill children already treated in a PICU with slightly more favourable survival rates [8, 11, 13]. Less than one-tenth of CPR events occur in the paediatric emergency department (PED), with a much poorer survival when compared to other in-hospital locations [14]. The underlying causes of paediatric CA show a high prevalence of respiratory and circulatory insufficiency, in 60 to 70% of the OHCA- and PED-patients CA is preceded by respiratory failure [8, 10, 13, 14].

Due to this high number of respiratory causes for CA in children, ventilation is prioritized in paediatric CPR [8, 15]. However, so far there is no data to support the assumption, that early endotracheal intubation (ETI) is vital in paediatric resuscitation. In fact, ETI might even be detrimental to CPR performance [16] and impair the neurological outcome [17, 18]. A potential lack of expertise in managing a paediatric airway promotes adverse events [19–22]. The current guidelines strongly suggest the use of bag-mask ventilation (BMV) as the primary method of paediatric airway management, as it is easy to learn and reliable [15]. Nevertheless, the initiation of ventilation in paediatric respiratory insufficiency is often delayed [6].

Simulation-based training (SBT) can improve procedural skills, team performance, and guideline adherence in paediatric emergencies and resuscitations [23–26]. Several studies demonstrated a positive effect of SBT on the quality of chest compressions, and the timely implementation of time-critical measures [24, 27–30]. However, little has been published regarding the effects of participation in SBT on airway management during paediatric resuscitation [26]. In our study we aim to (1) evaluate basic and advanced airway management during simulated paediatric resuscitations in children’s hospitals before and after a structured paediatric SBT, (2) measure the influences of airway management on other advanced life-support interventions, and (3) evaluate for differences in airway management between hospitals with and without a PICU.

## Methods

Between April 2017 and January 2018, standardised paediatric SBT was conducted in 12 of the 15 children’s hospitals in the German Federal State Hesse, supported by an initiative of the Hessian Ministry for Social Affairs and Integration (HMSI). Although each of these hospitals provides emergency care for critically ill children, there is a high variability in patient capacities (40 to 150 beds) and annual patient volume. Only six of these 12 children’s hospitals maintain a PICU (4 to 13 beds).

A prospective interventional study was performed in these 12 children’s hospitals to assess paediatric emergency care. The primary endpoints of the study were to assess adherence to guidelines in simulated paediatric cardiac arrests due to shockable rhythms and to assess teamwork and team communication in these cardiac arrests. The assessment instruments used are reported elsewhere [31]. A secondary analysis evaluated airway management to address the research questions outlined above.

Ethical approval was obtained from the Ethics Committee of the Philipps-University of Marburg (AZ: 172/16). Written informed consent was obtained from all study participants.

A detailed methodology report, based on the reporting guidelines for simulation-based research [32], is provided in Supplementary material 1: Appendix 1.

### Simulation-based training

**Fig. 1:**
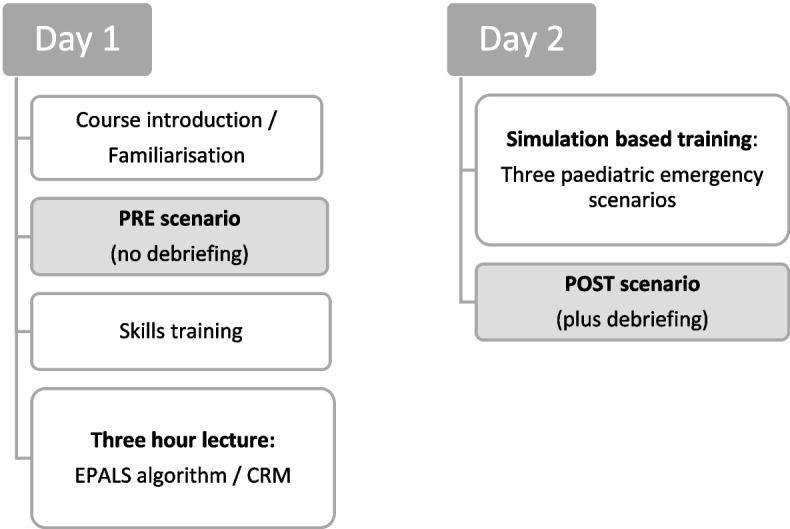
Study design

The SBT was standardised across all children’s hospitals and delivered as in-house training on two consecutive days. It consisted of a 3-h interactive lecture and three simulation scenarios (see Fig. 1). Two hours of the lecture focused on the recognition of critically ill children, paediatric basic and advanced life support (EPALS), including airway management, cardiac rhythm recognition, and shockable and non-shockable cardiac rhythm algorithms according to European Resuscitation Council guidelines [33]. Crisis resource management (CRM) aspects were covered in a further hour. Simulation scenarios were performed with high-fidelity mannequins (Gaumard HAL3010 tetherless newborn simulator and HAL3005 tetherless 5-year-pediatric simulator). These scenarios were scripted including specific learning objectives and consisted of a respiratory, a circulatory, and a neurological paediatric emergency leading to apnoea and cardiac arrest with a non-shockable cardiac rhythm (see Supplementary material 1: Appendix 1). Varying resuscitation teams of up to six participants took part in these simulation scenarios. Resuscitation teams were always interprofessional. Participants not taking part in a simulation scenario were able to watch via an audio–video system in a nearby room. Each simulation scenario was followed by a structured and scripted debriefing using the PEARLS framework [34]. Debriefing was conducted in an interprofessional team of one physician and one nurse, out of a total team of four physicians and five nurses, and lasted approximately 20 to 30 min. All physicians were consultants in paediatrics or anaesthesiology with at least 2 years of experience in paediatric intensive care. Nurses were paediatric intensive care specialists with many years of professional experience. All nine members of the research team had been formally trained as simulation trainers in a train-the-trainer course for simulation and had at least 2 years of debriefing experience.

Depending on the hospital’s preference, the training was conducted in PEDs, on inpatient wards, or PICUs, using the emergency medical equipment available on site. Participation in the SBT was voluntary and varied between hospitals, with the research team having no influence on participation rates.

The course was developed by experts in paediatric emergency medicine, paediatric intensive care, and simulation-based training. It was piloted in the PICU of the Department of Paediatrics at Philipps-University in Marburg, Germany.

### Study participants

Study participants were recruited from the SBT participants at each children’s hospital and included paediatric nurses and physicians with different levels of experience. No other professionals (e.g. respiratory therapists) or other specialties (e.g. paediatric anaesthesia) were included. Study participation was voluntary. There were no exclusion criteria for participation except for lack of consent.

Participants autonomously formed study teams of four people, including at least one nurse and one physician. The composition of the teams varied in each of the simulation and study scenarios to reflect the realities of working within ad hoc emergency teams.

Each study participant completed questionnaires about demographics and previous resuscitation experience.

### Study scenarios

Immediately before and after the SBT, study participants took part in two study scenarios (PRE and POST scenario, Fig. 1 and Supplementary material 1: Appendix 1), which were recorded using an audio–video system with three different camera angles. The PRE and POST scenarios differed only in the patient history provided to the teams but followed the same clinical progression of apnoea, cardiac arrest, and return of spontaneous circulation (ROSC) with identical vital signs.

Study scenarios were scripted to last 12 min regardless of the actions performed. A critically ill infant was presented to the study teams in the PED or in the paediatric ward. After two minutes, the simulated patient went into apnoea and cardiac arrest with a shockable cardiac rhythm. Eight minutes later, the patient had a ROSC regardless of the study team’s resuscitation interventions. ROSC could have been achieved earlier if the study team had performed the EPALS algorithm correctly (adequate CPR technique, three correct shocks, epinephrine, and amiodarone in the correct dose and at the correct time). The scenario was terminated two minutes after ROSC. The POST scenarios were followed by a structured debriefing, the PRE scenarios were not debriefed. Familiarisation with the simulator and orientation to the SBT environment was provided during the 60-min course introduction prior to the PRE scenario (see Fig. 1).

In contrast to the simulation scenarios, shockable cardiac rhythms were chosen for the study scenarios to avoid improvements in the POST scenarios being due solely to “familiarisation” with algorithms.

### Performance evaluation

To assess airway management, a performance evaluation checklist was developed by experts in paediatric intensive care and SBT through a two-stage Delphi process [35]. The performance evaluation checklist consisted of 27 items in three categories: basic airway management during cardiac arrest, evaluation of endotracheal intubation if performed, and timing of specific airway management (see Supplementary material 1: Appendix 1). A manual was developed to specify the rating of each item, and rater training was conducted using a previously published rater training programme [31] achieving high interrater reliability (intraclass correlation coefficient: 0.93). PRE and POST scenario videos were randomised by the principal investigator (NM), and analysed by a blinded rater not initially involved in data collection (CD) and highly experienced in paediatric intensive care and SBT.

### Sample size

A sample size of 41 teams was calculated using an effect size of 0.3 (medium effect size), with a type I error of 0.05 and power of 0.8 [36]. A similar study measuring the effects of SBT on adherence to PALS guidelines determined a sample size of 51 teams [37]. We aimed to recruit at least 50 teams.

### Data analysis

Data were analysed using IBM SPSS Statistics Version 29.0. Categorical variables were expressed as frequencies and percentages. Chi-square tests were used for comparing frequencies of categorical variables in PRE and POST scenarios. Arithmetic mean and standard deviation were used for characterizing initiating times e.g. time to ventilate, and unpaired t-tests for comparing PRE and POST scenarios. The level of significance was *p* < 0.05.

## Results

A total of 276 nurses and doctors completed the simulation-based training. 229/276 (83%) participants agreed to take part in the study and formed 58 PRE and 58 POST scenario study teams. After the exclusion of three videos due to poor audio quality, 56 PRE and 57 POST scenario videos were analysed. The participants’ professional roles and previous clinical experience are described in Table 1.

**Table 1:** Characteristics of study participants

**Professional and educational characteristics**	***n*/*N* (%) of cohort**
Professional role	
Head of department	2/229 (0.9)
Senior physician	19/229 (8.3)
Resident physician	81/229 (35.4)
ICU nurses	31/229 (13.5)
Other nurses	91/229 (39.7)
n/a	5/229 (2.2)
Years of experience^a^	0–43 years^b^
Previous experienced paediatric resuscitations^c^	
Senior physician	8.3^d^ (range 1–20)
Resident physician	3 (range 0–10)
Nurses	0.4 (range 0–6)
ICU nurses	3 (range 0–10)
Ventilation experience	
Senior physicians	8/19 (42.1)
Resident physicians	33/81 (40.7)
Nurses	20/91 (22.0)
ICU nurses	17/31 (54.8)
Previous resuscitation training	194/229 (84.7)
*Within last 12 months*	107/229 (46.7)

^a^Refers to the number of years worked in current role

^b^Range (minimum–maximum)

^c^Neonatal resuscitations were explicitly excluded

^d^Arithmetic mean

### Basic airway management

**Fig. 2:**
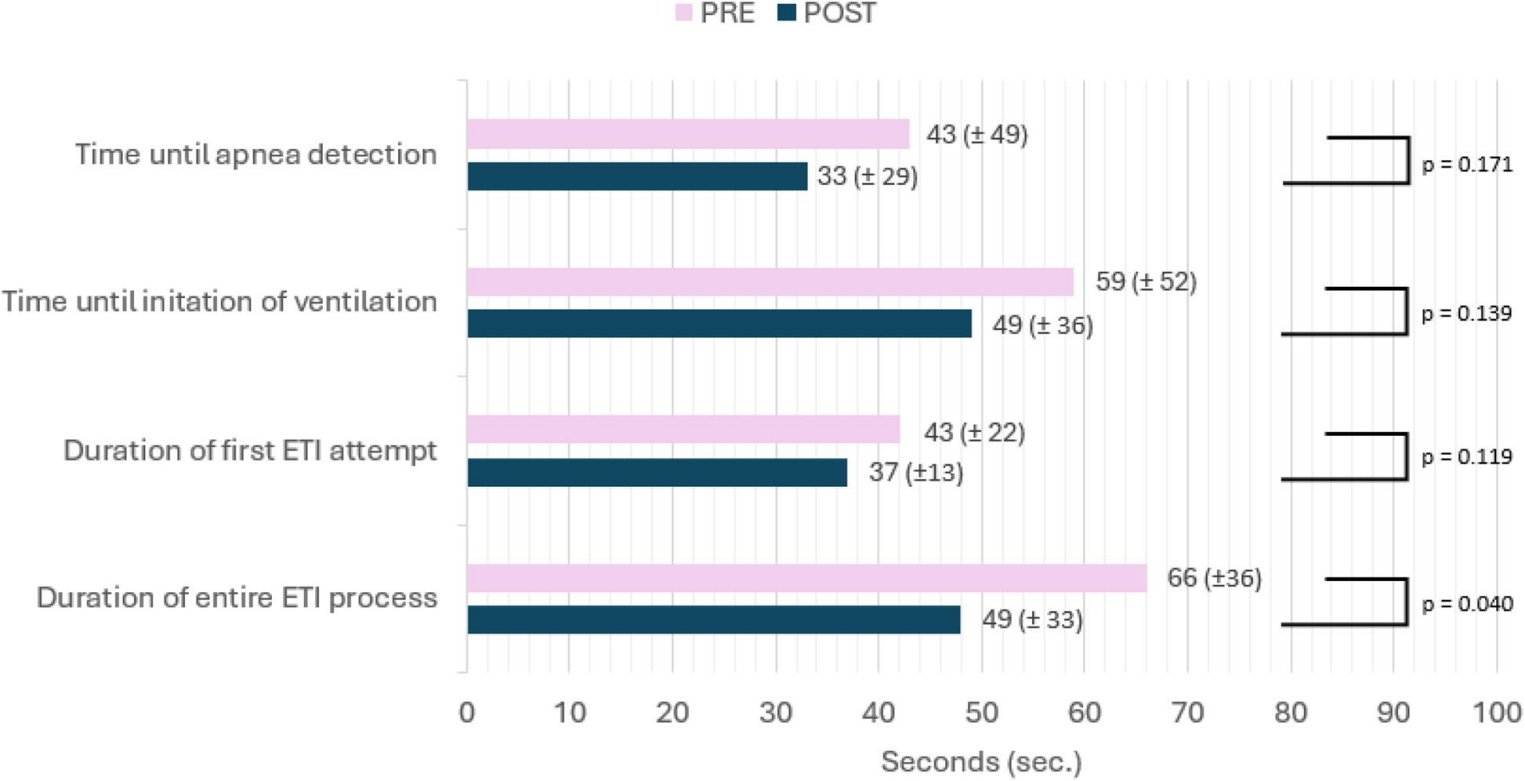
Timing of basic and advanced airway management before and after SBT

All teams recognised apnoea in their patients and initiated ventilation during the scenarios. Nurses were significantly more likely to recognise and communicate apnoea after SBT (PRE 27% vs. POST 50%, *p* = 0.018). Time to recognition of apnoea (PRE 43 s ± 49 s, CI95 26–59 s; POST 33 s ± 20 s, CI95 23–44 s; n.s.) and time to initiation of ventilation (PRE 59 s ± 53 s, CI95 44 – 74 s; POST 49 s ± 36 s, CI 40–59 s; n.s.) did not improve significantly between the PRE and POST scenarios (Fig. 2).

93% of PRE and POST teams used bag-mask ventilation (BMV) as the first line of ventilation. POST teams were significantly more likely to select appropriately sized BMV equipment (46% vs. PRE 65%, *p* = 0.048).

### Advanced airway management

Endotracheal intubation (ETI) was attempted in 55% PRE and in 40% POST scenarios (*p* = 0.10). More than one ETI attempt was necessary in 45% PRE and 30% POST scenarios (*p* = 0.27). On average, two to three participants were involved in the ETI process (PRE 2.6 ± 0.7 vs. POST 2.4 ± 0.7, n.s.). Appropriate equipment for ETI was used in 71% of the PRE and 95% of the POST scenarios (*p* = 0.075).

The duration of the entire ETI process was significantly shorter in the POST scenarios (Fig. 2). Auscultation of lung ventilation was chosen primarily to check for successful intubation, and end-tidal carbon dioxide was only measured in one PRE and one POST scenario, respectively.

### Influence of airway management on advanced life support interventions

**Fig. 3:**
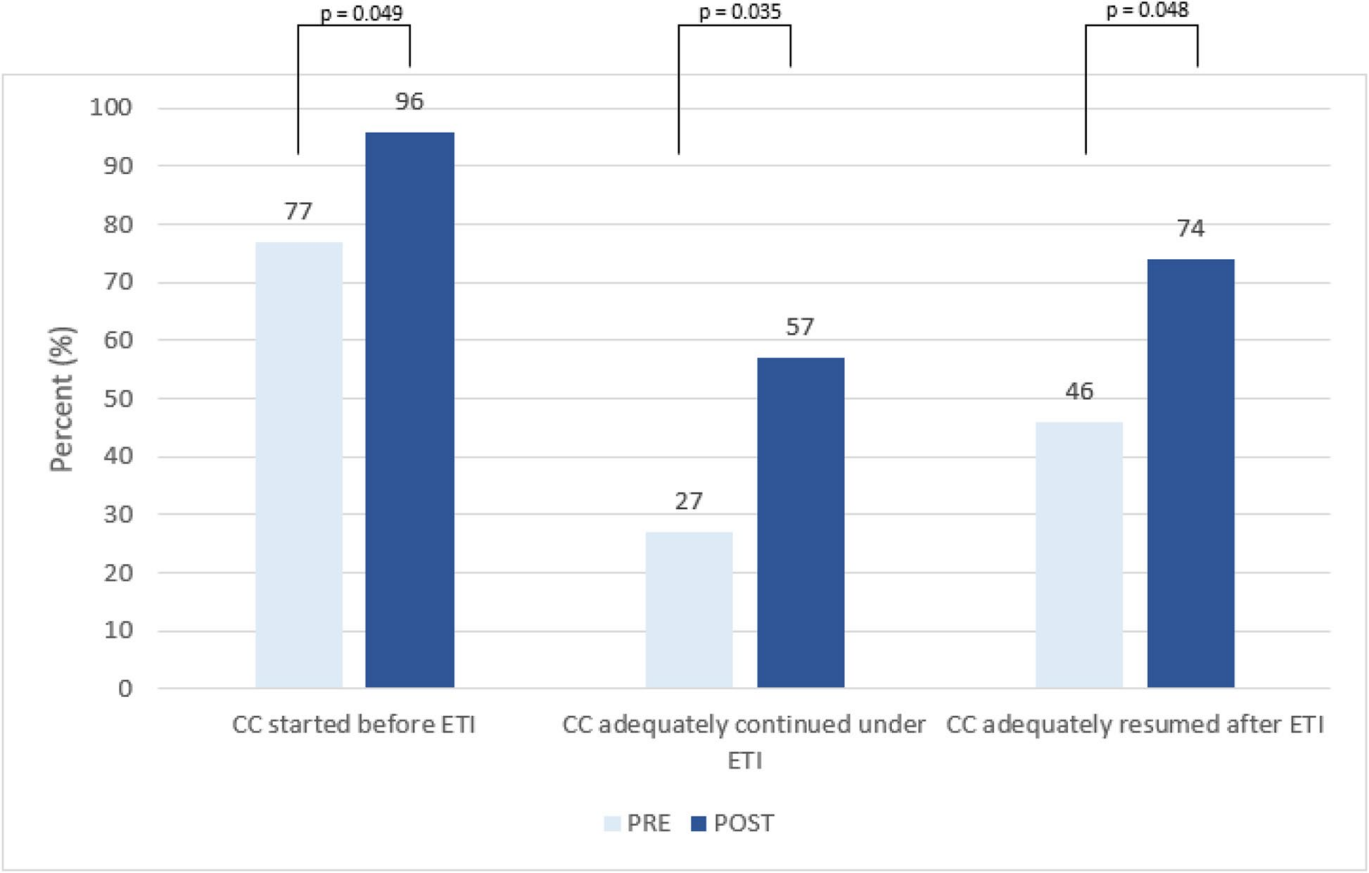
Quality of chest compressions (CC) before and after SBT

In 23% PRE and 4% POST scenarios, chest compressions (CC) had not been started at the time of the first ETI attempt (*p* = 0.049). CC were frequently discontinued during ETI attempts, which improved after the simulation training (PRE 73% vs. POST 43%, *p* = 0.035). Adequate resumption of chest compressions after completion of intubation was also significantly more frequent in the POST scenarios (46% vs. 74%, *p* = 0.048) (Fig. 3).

Initiation of defibrillation was frequently delayed during ETI attempts (85% vs. 65%, n.s.).

### Differences in airway management in relation to the maintenance of a PICU

29/56 (52%) PRE scenario videos and 30/57 (51%) POST scenario videos originated from hospitals with a PICU. Time to detect apnoea (PICU 32.4 ± 30.9 s, CI95 22–43 s; NON-PICU 44.1 ± 47.4 s, CI95 28–60 s; n.s.) and to initiate ventilation (PICU 49.1 ± 34.5 s, CI95 40–58 s; NON-PICU 59.7 ± 53.2 s, CI95 44–75 s; n.s.) did not differ significantly between both groups (combined PRE and POST data). Before SBT, 62% of teams from hospitals with a PICU vs. 48% of teams from hospitals without a PICU attempted ETI at least once. After SBT, 47% and 33% of the teams, respectively, attempted ETI (*p* = 0.20).

The number of teams that never performed chest compressions (CC) was significantly lower in hospitals with a PICU (PRE scenarios: PICU 13% vs. NON-PICU 36%; POST scenarios: PICU 0%, NON-PICU 11%; *p* < 0.01). During ETI attempts, CC were more likely to be adequately continued in teams from hospitals with a PICU (PRE scenarios: PICU 20% vs. NON-PICU 36%; POST scenarios: PICU 79%, NON-PICU 22%; *p* < 0.01).

## Discussion

We evaluated airway management in simulated paediatric resuscitations before and after SBT in children's hospitals in Hesse, Germany. 58 study resuscitation teams comprising 229 nurses and physicians from 12 out of 15 children’s hospitals participated in a structured two-day simulation-based training (SBT). Previous experience and expertise varied widely as participants originated from paediatric emergency departments, paediatric intensive care units, and paediatric general wards representing a wide spectrum of paediatric healthcare professionals in Hessian inpatient care. Airway management changed following a 2-day SBT intervention, leading to a reduction in interruptions of chest compressions during simulated paediatric resuscitations. Translation into the clinical context could improve the quality of resuscitation, resulting in better patient outcomes.

Even before SBT, ventilation was initiated by all resuscitation teams, suggesting that all participants were aware of its importance. The time to initiate ventilation varied widely, with teams starting ventilation after an average of 60 s in the PRE scenarios. The interval was not significantly reduced after SBT. This is consistent with data from Hunt et al. and Roy et al. where ventilation in paediatric emergency patients was rarely initiated within one minute of apnoea [4, 6, 38]. However, we found SBT had an impact on the recognition and communication of apnoea, thus demonstrating the successful involvement of all team members in the mental model of the critically ill child. This has been shown to promote vigilance of all team members to changes in the clinical situation which can ultimately lead to a better team performance [39–41].

Due to the heterogeneous origins of the paediatric healthcare professionals in our study cohort, experience with ventilation was highly variable. Two-thirds of all participants reported little or no experience in ventilating a child in an emergency setting before this training. This lack of experience is reflected in the initial poor choice of appropriate equipment. Although bag-mask ventilation was chosen as the primary ventilation mode in more than 90% of simulated resuscitations, more than 50% of resuscitation teams used incorrectly sized masks and ventilation bags. Current guidelines strongly suggest the use of bag-mask ventilation as the primary method of airway management as it is easy to learn and reliable [15]*.* However, difficulties in selecting appropriate equipment have been described before and as the use of incorrectly sized equipment may diminish the quality of ventilation it may harm the performance in paediatric resuscitation [21, 22, 42, 43]. SBT significantly improved the likelihood of selecting appropriately sized BMV equipment in our study cohort.

Despite the limited experience of ventilating a critically ill child, more than half of the resuscitation teams attempted endotracheal intubation (ETI) in the PRE scenarios. Even teams from hospitals without a PICU attempted ETI at least once in 48% with only half of the teams succeeding on the first attempt. Those low first-pass success rates have been observed in paediatric emergency intubations [19, 44–47] and are particularly alarming, as ETI in an emergency setting, especially when performed by non-experts, holds a high risk of adverse events like hypoxia, hypotension or aspiration [20, 44, 45, 48, 49]. In addition, ETI attempts in our cohort were time and personnel-consuming, with ventilation being stopped during the ETI attempt, which has been shown to prolong hypoxia and potentially worsen the outcome [18, 50]. Inappropriate ETI equipment and a lack of assessment of correct ETI placement further contribute to the risk of adverse events [49].

We were able to demonstrate that participation in SBT changed advanced airway management. Although the frequency of ETI attempts was not significantly lower after SBT (*p* = 0.1), intubation times decreased, thus reducing no-ventilation time. Although we could not show a reduction in people involved in the intubation process, the number of teams with proper ETI preparation increased to almost 100%.

Improving advanced airway management had a strong effect on other advanced life support measures. Before SBT, chest compressions were frequently discontinued during ETI attempts and inappropriately or not resumed after ETI. Long-term survival after cardiac arrest, and in particular survival with little or no neurological impairment, requires not only optimal airway management but also high-quality circulatory support. This means that chest compressions should be delivered with sufficient depth and frequency to ensure good cerebral perfusion. It is particularly important to minimize the number and duration of interruptions in compressions [51–54]. Donoghue et al. demonstrated significant interruptions in CC when ETI was attempted during paediatric CPR in their paediatric emergency department and found worse survival for patients with an invasive airway. They concluded that paediatric patients with CA benefited from withholding ETI attempts [14]. Wang et al. reported similar findings with frequent and prolonged pauses for intubation during paediatric resuscitation in the prehospital setting [55]. In our cohort, SBT resulted in less discontinuation of CC during intubation and adequate resumption after intubation. Thus, reducing the complexity of airway management may directly improve circulatory support.

The effect of ETI attempts on circulatory support is particularly noteworthy in hospitals with a PICU. Though those resuscitation teams were not significantly faster in detecting apnoea and initiating ventilation they attempted to intubate more frequently with high rates of interrupted chest compressions during that process. This highlights the necessity of different teaching approaches for different groups of healthcare professionals. While nurses and physicians working primarily on paediatric wards can be reassured that high-quality bag-mask ventilation is ‘good enough’ for paediatric resuscitation, paediatric intensive care teams need to be reminded to deliver high-quality chest compressions without interruption by ETI attempts. However, studies on paediatric airway management in hospitals with and without PICUs are lacking. Most studies compare the performance of resuscitation teams in single-centre trials or select participants from similar workplaces in their multi-centre trials, none compare resuscitation performance between hospitals maintaining a PICU and hospitals of primary care. Auerbach et al. compared the performance of paediatric resuscitation in paediatric emergency departments of centres with different annual patient volumes and concluded that the best guideline adherence in paediatric basic life support was in centres with medium–high patient volumes [29]. PICUs were not explicitly reported. Our study thus contributes to a better understanding of paediatric emergency care across different healthcare professional teams.

### Strengths and limitations

This is the first study to systematically investigate airway management in German children’s hospitals in a defined federal state. Healthcare professionals from 12 different children’s hospitals participated, including university hospitals and urban and suburban hospitals with different annual patient volumes, representing 80% of inpatient care in Hesse. Resuscitation teams were interprofessional and recruited from PICUs, paediatric emergency departments, and general paediatric wards, resulting in a wide range of previous experience and expertise among participants. This high variability among participants may have been the reason why we did not measure significant improvements in all items investigated after SBT. The observed airway management may also be influenced by the simulation setting, as participants felt less hesitant to intubate a mannequin than a real child [4, 18, 56]. As the care of the critically ill child rather than specific airway management was the primary goal of investigating potential effects of SBT and high-fidelity mannequins were used to enhance immersion in the simulated paediatric resuscitation, we believe that our results provide a good overview of actual airway management in paediatric hospitals.

Our study design may also have influenced the results. As we compared the performance of the PRE and POST teams in multiple hospitals with different annual patient volumes, and the composition of the PRE and POST teams in each hospital varied, a multi-level analysis was not considered appropriate. We did not evaluate which aspects of our SBT specifically led to the observed changes in airway management and whether lecture and skills training alone would have been sufficient for these. We conducted our SBT within suggested frameworks for high-quality SBT, such as simulation trainer qualifications, effective learning and simulation environments, and the process of scenario development and implementation, including standardised debriefing [57], to provide standardised learning conditions for trainees and to ensure lasting effects. However, it was not investigated how long the observed effects lasted and to what extent they were transferred to the clinical context.

## Conclusion

We demonstrated that airway management changed following a 2-day SBT intervention, resulting in a significant improvement in the quality of chest compressions during simulated paediatric resuscitations. To enhance the demonstrated effect, airway management should be targeted more explicitly in simulation-based training. In addition, the observed changes in airway management and its effect on other life support measures differed between healthcare teams in hospitals with and without PICUs. SBT may be adapted to the participants’ workplace to maximize its effect and improve the overall performance in paediatric resuscitation.

## Supplementary Information

Supplementary Material 1.

## Data Availability

No datasets were generated or analysed during the current study.
