# Supplementary material for: To intubate or to resuscitate: the effect of simulation-based training on advanced airway management during simulated paediatric resuscitations
Source: Adv Simul (Lond). 2025 Jan 6;10:1. doi: 10.1186/s41077-024-00326-y (PMC11705721; doi:10.1186/s41077-024-00326-y)

**Appendix:**

1. SBT methodology based on Cheng et al. [1]

| **Participant orientation** | **Orientation to the simulator** | Familiarisation with the simulator and orientation to the SBT environment was provided during a 60-minute course introduction prior to the PRE scenario. Simulator familiarisation was structured, scripted, and conducted using the ABCDE approach. Participants were made aware of the capabilities and limitations of the simulator. |
| --- | --- | --- |
|  | **Orientation to the environment** | As the training was conducted in different settings depending on the hospital's preference, on site emergency medical equipment was used. Participants had the opportunity to check this equipment. |
| **Simulator type** | **Simulator make and model** | Gaumard HAL3010 tetherless newborn simulator and HAL3005 tetherless 5-year-pediatric simulator were used for the SBT. In PRE and POST scenarios, the Gaumard HAL3010 tetherless newborn simulator was used exclusively. |
|  | **Simulator functionality** | Simulators were high-fidelity patient simulators. A sternotomy scar was added to the neonatal simulator for one study scenario. No other specific modifications were made to the simulator.  In both simulators, normal and abnormal breathing including unilateral or bilateral chest rise is visible, bag-mask ventilation, airway adjuncts and nasal or oral intubation are possible, lung sounds are detectable. Heart sounds can be auscultated, chest compressions generate palpable blood pressure and central cyanosis is visible. There are IV and IM injection sites and an intraosseous access at the tibia. Seizures/convulsions are visible in both simulators. An active pupillary light reflex is visible in the junior simulator. |
| **Simulation environment** | **Location** | Training was conducted as an in-house training. Depending on the hospital's preference, the training was conducted in PEDs, inpatient wards, or PICUs. |
|  | **Equipment** | Teams used the on-site emergency medical equipment (PED, inpatient ward, PICU). The researchers did not influence the type, amount, location, or size of medical equipment. |
|  | **External stimuli** | Researchers did not apply specific external stimuli, e.g. background noise. |
| **Simulation scenarios** | **Event description/ learning objective** | Simulation scenarios were scripted and included specific learning objectives (see b) simulation scenario scripts).  The scenarios consisted of a respiratory, a circulatory, and a neurological paediatric emergency leading to apnoea and cardiac arrest with a non-shockable cardiac rhythm. |
|  | **Group vs. individual practice** | Composition of resuscitation teams varied, up to six participants took part in the simulation scenario. Teams were always interprofessional. Participants not taking part in a simulation scenario were able to watch via an audio-video system in a nearby room. |
|  | **Use of adjuncts** | None. |
| **Study scenarios** | **Event description/ learning objective** | Study scenarios were scripted, and the clinical progression was programmed (see c) study scenario scripts). The PRE and POST scenarios differed only in the patient history provided to the teams but followed the same clinical progression of apnoea, cardiac arrest, and return of spontaneous circulation (ROSC) with identical vital signs.  Study scenarios were scripted to last 12 minutes regardless of the actions performed. A critically ill infant was presented to the study teams. After two minutes, the simulated patient went into apnoea and cardiac arrest with a shockable cardiac rhythm. Eight minutes later, the patient had a ROSC regardless of the study team's resuscitation interventions. ROSC could have been achieved earlier if the study team had performed the EPALS algorithm correctly (adequate CPR technique, three correct shocks, epinephrine, and amiodarone in the correct dose and at the correct time). The scenario was terminated two minutes after ROSC. |
|  | **Group vs. individual practice** | The composition of study teams varied between PRE and POST scenarios. Participants autonomously formed study teams of four people, including at least one nurse and one physician.  Participants not taking part in the study scenario were not able to watch those study scenarios. |
|  | **Use of adjuncts** | A sternotomy scar was added to the neonatal simulator for one study scenario. No other specific modifications were made to the simulator. |
| **Human resources** | **Facilitator/operator characteristics** | Four physicians, five nurses and three technicians were part of the simulation trainer team in this study. Each simulation training session was conducted with two medical staff (research team) and one technician. The research team was always interprofessional, with a physician and a nurse conducting the briefing, training and debriefing.  All physicians were consultants in paediatrics or anaesthesiology with at least two years' experience in paediatric intensive care. Nurses were paediatric intensive care specialists with many years of professional experience.  All four physicians and four of the five nurses were current EPALS providers. All nine members of the research team had been formally trained as simulation trainers in a train-the-trainer course for simulation and were familiar with debriefing.  To standardise SBT in all children´s hospitals, all research team members were formally briefed for this study in a one-day course. Lecture content, simulation and study scenarios, and debriefing method using the PEARLS framework were thus standardised. |
|  | **Pilot testing** | The course was piloted as a two-day-course in the PICU of the Department of Paediatrics at Philipps-University in Marburg, Germany. |
|  | **Actors/simulated patients** | In each simulation and study scenario, at least one member of the research team acted as a paramedic, nurse or parent to describe the simulated patient to the participants. No other actors or simulated patients were involved. |

| **Instructional design** | **Duration** | 1. Course introduction and familiarisation: 60 min 2. PRE scenarios: approx. 60 min 3. Skills training: 60 min 4. Lecture on EPALS algorithm: 120 min   Lecture on Crisis resource management: 60 min   1. Three simulation scenarios: 210 min 2. POST scenarios: approx. 60 min 3. Debriefing POST scenarios: approx. 30 min |
| --- | --- | --- |
|  | **Timing** | Study scenarios were conducted immediately before and after SBT. The whole course was conducted as a two-day course. |
|  | **Frequency/repetitions** | Simulation and study scenarios could not be repeated. |
|  | **Clinical variation** | Study scenarios differed in patient history provided to the teams. Initial vital parameter and clinical progression were identical (see “Event description of study scenarios”). The order of study scenarios varied in the children’s hospitals. |
|  | **Standards/assessment** | The study scenarios followed a predefined structure. Within the first two minutes, the teams were expected to assess the critically ill child, administer oxygen, call for help and communicate the compensated shock. After two minutes, the simulated patient went into apnoea and cardiac arrest with a shockable cardiac rhythm. Participants were expected to initiate bag-mask ventilation and CPR, deliver three shocks, and administer epinephrine and amiodarone at the correct dose and time. Eight minutes after apnoea, the patient had ROSC regardless of the study team's interventions. During ROSC, participants were expected to reassess the patient and plan further actions to stabilise the patient.  The study scenarios were recorded using an audio-video system. To retrospectively assess airway management, a performance evaluation checklist was developed by experts in paediatric intensive care and SBT through a two-stage Delphi process, consisting of 27 items in three categories (see Appendix C).  The evaluation of the videos was blinded and randomised. All videos were analysed by an observer who was not involved in the SBT, following specific rater training. |
|  | **Adaptability of intervention** | The SBT structure was not customised. However, children´s hospitals decided autonomously where SBT would take place (PED, inpatient ward or PICU). As a result, participants and study teams had varying or no experience of paediatric emergency or intensive care. |
|  | **Range of difficulty** | Simulation scenarios within the SBT became increasingly complex, starting with status epilepticus and benzodiazepine overdose, through hypovolemic shock in a patient with gastroenteritis, to a patient following near drowning.  Study scenarios differed in patient history, but not in complexity. |
|  | **Nonsimulation interventions and adjuncts** | Two hours of the three-hour lecture focused on the recognition of critically ill children, paediatric basic and advanced life support (EPALS), including airway management, cardiac rhythm recognition, and shockable and non-shockable cardiac rhythm algorithms according to ERC guidelines. Crisis resource management (CRM) aspects were covered in a further hour. |

| **Feedback and/or debriefing** | **Source / Duration** | Debriefing was conducted by the interprofessional research team after each simulation scenario and lasted approximately 20 to 30 minutes. |
| --- | --- | --- |
|  | **Facilitator presence / characteristics** | Each debriefing was conducted in an interprofessional team of one physician and one nurse, out of a total team of four physicians and five nurses. All physicians were consultants in paediatrics or anaesthesiology with at least two years' experience in paediatric intensive care. Nurses were paediatric intensive care specialists with many years of professional experience.  All nine members of the research team had been formally trained as simulation trainers in a train-the-trainer course for simulation and were familiar with debriefing. |
|  | **Content / structure / method / scripting** | Debriefing method was standardised and scripted using the PEARLS framework. Debriefing focused on guideline adherence (recognition of apnoea and cardiac arrest, initiation of basic life support, recognition of cardiac rhythm and initiation of epinephrine or defibrillation) and crisis resource management (teamwork, communication). |
|  | **Timing** | Debriefing was conducted immediately after each simulation scenario and after all POST scenarios. The PRE scenarios were not debriefed. |
|  | **Video** | The debriefing of simulation scenarios could be supported by video debriefing if requested by the participants. Video debriefing was not routinely used in every debriefing session.  Video was not used in the debriefing of POST scenarios. |

1. Cheng, A., et al., *Reporting Guidelines for Health Care Simulation Research: Extensions to the CONSORT and STROBE Statements.* Simul Healthc, 2016. **11**(4): p. 238-48.

1. Simulation scenario scripts

| **Status epilepticus** | |
| --- | --- |
| **Learning objectives** | Medical: Treatment algorithm for status epilepticus; alternative methods of drug administration; airway management. NTS: Team coordination, anticipation of respiratory decline after benzodiazepine administration. |
| **Scenario description** | Admission of a 4-year-old boy by paramedics after a seizure with fever. The child had suddenly become unresponsive at home, with gaze deviation, hypotonic muscle tone and tongue fasciculation. The parents administered diazepam 10 mg supp. On arrival of the paramedics, clonic activity in both legs, therefore repeat dose of diazepam (10mg supp.) and termination of the seizure. Paracetamol (250mg supp), oxygen (8 L/min), and transfer to the children's hospital without complications.  AMPLE: Two seizures with fever, 6 and 18 months ago. EEG unremarkable. No other previous illnesses/allergies. Weight: approximately 15kg |
| **Checklist for scenario preparation** | Simulator: Junior  Required props: Simulator is fully clothed, no IV, oxygen via nasal cannula. The simulator is lying sideways on a medical carrier.  (Additional) Medical equipment: Mucosal atomization device (MAD), airway adjuncts  Human resources: two instructors (an emergency doctor, bringing the simulated patient and a parent, accompanying the patient) |
| **Participant briefing** | The scenario takes place in the PED, an emergency doctor brings the patient in and gives the patient’s history. |
| **Planned scenario sequence** Initial vital parameters | HR: 146/min, BP: 109/57 mmHg; SpO_2_ 82%; RR 20/min, cyanosis 40%  *If asked:* temperature 39,4°C, RCT 3 sec  Child is drowsy and shows clonic activity in one arm, breathing is labored. Blood sugar 131mg/dl. |
| Aim | - Recognition of acute seizure, administration of medication - Algorithm for drug administration in status epilepticus - Anticipation of or deterioration with multiple benzodiazepine administration (have the BMV ready!) - Recognizing labored breathing: airway adjuncts |
| Scenario progression | Airway management   - Open airway, consider airway adjuncts, switch to reservoir mask: SpO_2_ ↑ max. 92% - Otherwise SpO_2_ slowly drops to 70% - Circulation **stable** at all times   Seizure management   - MAD/buccal administration of benzodiazepine: SpO_2_ drops to at least 64%, seizure idem - IV administration of benzodiazepine: seizure stops, but apnoea and SpO_2_ 43% → BMV - Consider second line medication |
| Life saver | Seizure stops. |

| **Hypovolemic shock** | |
| --- | --- |
| **Learning objectives** | Medical: Recognition and adequate treatment of paediatric shock; alternative injection sites (IO); algorithm non-shockable rhythm. NTS: Role allocation, team coordination. |
| **Scenario description** | 4-year-old patient admitted to the children’s hospital the day before with rotavirus. He has had a fever of up to 40°C, watery diarrhoea and vomiting for 3 days. The establishment of a new intravenous access has proven difficult in the morning. The patient has deteriorated during the day and is admitted to the PICU in decompensated hypovolemic shock. AMPLE: unremarkable pregnancy and birth, unremarkable development to date. No previous illnesses/allergies. Weight: 18kg |
| **Checklist for scenario preparation** | Simulator: Junior  Required props: Simulator is fully clothed, no IV. The simulator is lying in a bed.  (Additional) Medical equipment: EZ-IO  Human resources: two instructors (a nurse, bringing the simulated patient and a parent, accompanying the patient) |
| **Participant briefing** | The scenario takes place in the PICU, a nurse brings the patient in. The nurse gives the patient’s history. |
| **Planned scenario sequence** Initial vital parameters | HR: 165/min, BP: MAD 50mmHg; SpO_2_ 93%; RR 42/min, cyanosis 10%  *If asked:* temperature 39,7°C, RCT 6 sec  Child is drowsy, shows little spontaneous activity. Peripherals are cool, mottled. Blood sugar 67mg/dl. |
| Aim | - Recognition of decompensated shock and adequate fluid management (crystalloid, 3 x 20 ml/kg in 15min, always re-evaluation) - Intraosseous access |
| Scenario progression | - Intravenous access not possible - Child continues to deteriorate, bradypnoea: RR 8/min, SpO_2_ 52% → BMV required. - HR ↓ drops to 40/min → start CPR - With efficient resuscitation/volume management (20ml/kgKG crystalloid volume at least once): HF increases to max. 84/min - Clinical improvement (awake, RCT 2 sec, normal BD / HR) after a total of 3 volume boluses - With ineffective resuscitation: persistent bradycardia |
| Life saver | During prolonged resuscitation, the heart rate increases without volume administration with an adequate dose of adrenaline. |

| **(Near) drowning** | |
| --- | --- |
| **Learning objectives** | Medical: algorithm non-shockable rhythm; differential diagnoses for respiratory deterioration after intubation (DOPES); airway management NTS: team coordination and communication |
| **Scenario description** | 9-month-old infant after near drowning. The infant had been left unattended in the bathtub for a few minutes; when the parents returned, they found the infant submerged. Bystander CPR (mouth-to-mouth resuscitation) started by the father under the guidance of the dispatcher, emergency medical services present after 5 minutes. Oral intubation by paramedics. Bag-mask ventilation with an adult bag and transfer to a children's hospital 5 minutes away. Circulation stable, SpO_2_ 94%, no sedation.  Infant is naked and wrapped in wet towels, orally intubated, with high-volume BMV. When transferring the patient from the stretcher, the tube is dislocated (unnoticed and uncommented). AMPLE: Last meal (evening porridge) 1 hour before. Unremarkable pregnancy and birth, unremarkable development to date. No previous illnesses/allergies. Weight: 6800 g |
| **Checklist for scenario preparation** | Simulator: Infant  Required props: Infant is naked, wrapped in wet towels. Orally intubated with a 3.0 tube, 'provisionally' fixated with a thin white tape. No IV access. SpO_2_ sensor on the patient, but disconnected.  (Additional) Medical equipment: adult ventilation bag  Human resources: two instructors (an emergency doctor and a paramedic, bringing the simulated patient. No parent.) |
| **Participant briefing** | The scenario takes place in the PED, an emergency doctor and a paramedic bring the patient in and give the patient’s history. No parents involved. |
| **Planned scenario sequence** Initial vital parameters | HR: 92/min, BP: 65/42 50mmHg; SpO_2_ 94%; RR 12/min, cyanosis 10%, CO_2_ 22  *If asked:* temperature 35,2°C, RCT 4 sec  Infant is unconscious, no spontaneous activity. Peripherals are cool, mottled. Blood sugar 153mg/dl. |
| Aim | - Apply DOPES in case of deterioration: Recognise incorrect intubation and gastric hyperinflation - Initiate adequate ventilation (BMV or re-intubation), monitor adequate ventilation (CO_2_) - Select the correct tube size/ventilation bag - Thermal management |
| Scenario progression | - After (unnoticed) tube dislocation caused by the emergency physician, SpO_2_ drops slowly to 67 %; HR drops slowly to 53 bpm → CPR - With adequate processing of the DOPES (establishment of mask ventilation or re-intubation, gastric tube or deep suction), rapid improvement of the HF and SpO2 (max. 1 cycle HDM) - If the problem is not recognized, bradycardia persists - In the absence of thermal management, increasing hypothermia and hypoxemia (min. SpO_2_ 84%) |
| Life saver | The infant cries to demonstrate the dislocated tube. |

1. Study scenario script

| **PRE / POST scenario** | |
| --- | --- |
| **Learning objectives** | Medical: EPBLS, EPALS: algorithm shockable rhythm; NTS: team coordination and communication |
| **Scenario description** | **I:** The emergency physician brings a 1-year-old female infant with unclear loss of consciousness. She has had severe watery diarrhea and recurrent vomiting for 2 days. Food refusal, anuria. She hasn't woken up since her afternoon nap. The emergency physician administered diazepam 5mg supp once for suspicion of seizures. Blood sugar was normal. Vital signs are stable during transport. Approx. 2 minutes after initial evaluation in the PED, she develops ventricular fibrillation.  AMPLE: Last feed about 3 hours ago. Unremarkable pregnancy and birth, unremarkable development to date. No previous illnesses/allergies. Weight: approximately 10 kg. **II:** Direct presentation in the PED. 6-month-old female infant with a corrected heart defect. During the previous week, she had symptoms of a cold, in the last 2 days increasing shortness of breath, vomiting when coughing and refusal to eat, elevated temperature up to 38.8 °C. Increasingly lethargic in the last 2 hrs, sweating profusely, looking very ill and grey. Approx. 2 minutes after the initial assessment, she develops a broad complex tachycardia. AMPLE: Last feed day before. No allergies. Cardiac surgery with 3 months. Weight: approximately 6 kg.  The emergency physician (I) and the parent (II), respectively, give a structured patient history. **Final sentence: “The infants seems very sick to me.”** |
| **Checklist for scenario preparation** | Simulator: Infant  Required props: Infant is dressed, parent is carrying the infant in the PED. For II: sternotomy scar  (Additional) Medical equipment: defibrillator, pads.  Human resources: two instructors (an emergency doctor and a parent.) |
| **Participant briefing** | The scenarios takes place in the PED, an emergency doctor and a parent bring the patient in. Structured patient history giving by emergency doctor (I) or parent (II). |

| **Planned scenario sequence** Initial vital parameters  (both scenarios!) | HR: 190 bpm (SR), BP: 60/20 mmHg; SpO_2_ 94%; RR 60/min, cyanosis 0%  *If asked:* temperature 36,0°C, RCT 4 sec, drowsy |
| --- | --- |
| Scenario progression | 1. **Compensated shock, 2 min**  - Vital parameters as above  1. **Shockable cardiac rhythm, max. 8 min**  - Apnea, SpO_2_ drops to 53%. Shockable rhythm, no pulse, no life signs - Sinus rhythm occurs after 8 min regardless of team intervention - Sinus rhythm occurs before 8 min when the team establishes adequate CPR, administers 3 correct shocks and meds (epinephrin and amiodaron) at correct time in correct dose  1. **ROSC, 2 min**  - Hr 180 bpm (SR), BD 70/20 mmHg, RR 10/min, SpO_2_ 90% - unconscious, RCT 4sek |
| Life saver | none |

1. Performance evaluation checklist


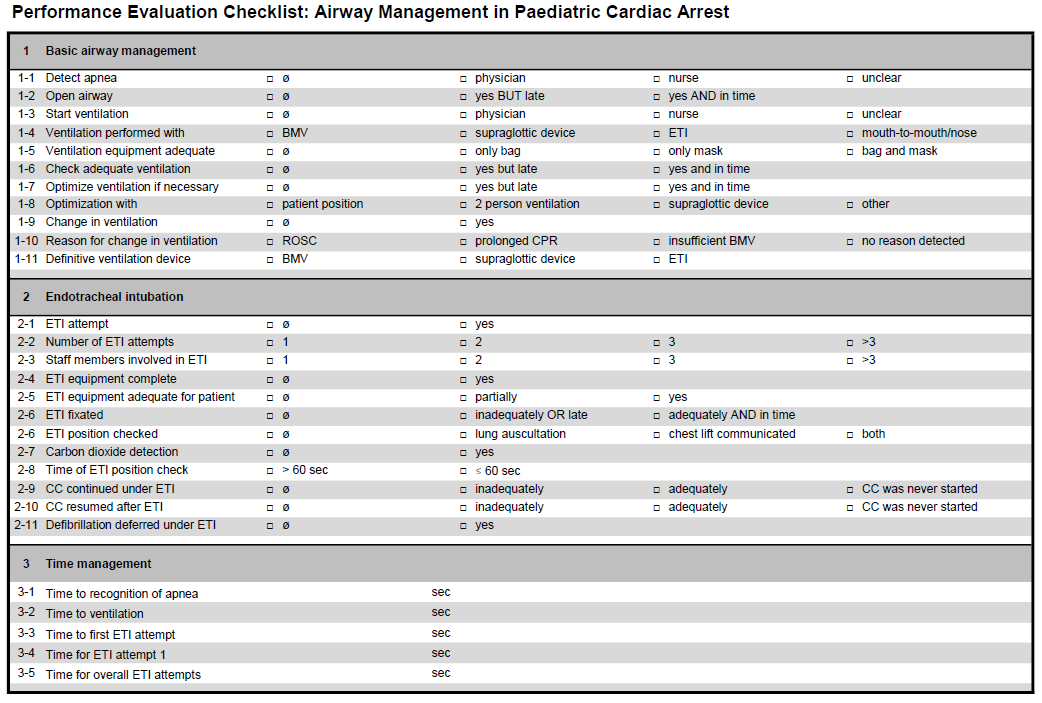

Supplement: Supplementary file 1 — Supplementary Material 1. [file 41077_2024_326_MOESM1_ESM.docx]
